# Dealing with Childhood Externalizing Behavior: Protocol for a Feasibility RCT of the Being a Parent Program

**DOI:** 10.3390/healthcare13020176

**Published:** 2025-01-17

**Authors:** Laura Maciel, Ana Rita Pires, Miguel Basto-Pereira, Crispin Day

**Affiliations:** 1 William James Center for Research, Ispa-Instituto Universitário, 1149-041 Lisboa, Portugal; anaritaf_pires@hotmail.com (A.R.P.); miguelbastopereira@hotmail.com (M.B.-P.); 2 Department of Psychology, Child & Adolescent Mental Health Service Research Unit, King’s College London, Institute of Psychiatry, Psychology and Neuroscience, London SE5 8AF, UK; crispin.1.day@kcl.ac.uk; 3 Centre for Parent and Child Support, South London and Maudsley NHS Foundation Trust, Michael Rutter Centre, London SE5 8AZ, UK

**Keywords:** parenting intervention, childhood externalizing behavior, randomized controlled trial, feasibility, protocol

## Abstract

Concerning numbers of childhood behavior problems have been reported worldwide. Parenting interventions are considered one of the most effective early strategies to intervene with externalizing conduct. This protocol outlines a feasibility RCT that aims to implement a parenting intervention in Portugal and (a) test key feasibility parameters, (b) assess the fidelity and acceptability of the program, and (c) explore its effectiveness in childhood behavior problems, parenting skills, parental concern, and parental sense of competence. A double-blinded, two-arm feasibility RCT is described. The participants are the primary caregivers of children between the ages of two and eleven years old that identify difficulties in managing their child’s behavior. Families will be randomly assigned to an intervention arm and a waitlist control arm. Parents in the intervention arm will participate in the Being a Parent program (Portuguese version: Ser Pai & Ser Mãe), an eight-week group intervention. Outcomes will be assessed using quantitative and qualitative measures throughout three assessment periods (pre- and post-intervention, and follow-up). This study marks the first feasibility RCT of the Being a Parent program outside the UK. The findings will provide information on the global strength of this program. Challenges and clinical implications are also discussed.

## 1. Introduction

Worldwide prevalences present concerning numbers of childhood behavioral problems [1,2,3,4]. In most cases, these difficulties may arise as a child’s response to coping with multisystemic strains (e.g., adverse childhood experiences) and/or as the first signs of early diagnosis of emotional and behavioral disorders, such as Conduct Disorders (CDs). Simultaneously, they often manifest as predictors of youth’s future social, physical, and mental health issues and are especially linked to adverse outcomes such as school dropout, substance abuse, and criminal offending [5,6,7].

In this context, many stakeholders have been working extensively to globally counteract childhood behavior problems and, consequently, the onset of a lifetime pathway of psychosocial maladjustment. The current empirical evidence strongly suggests that parenting interventions are one of the most cost-effective strategies to prevent and intervene with childhood behavior problems [8,9,10]. Their versatility and applicability across various developmental stages allow for the enhancement of parent–child relationships and the promotion of short- and long-term outcomes [11].

Consequently, it is no surprise that many international organizations [12] recommend the dissemination of evidence-based parenting programs around the globe. Taking into account that creating an intervention from scratch can have several associated costs [13], transporting evidence-based programs to other countries can be one of the most viable solutions for providing a cost-effective strategy to families. In fact, two recent meta-analyses examined the effectiveness of disseminating parenting interventions to other cultural contexts and concluded that interventions can still be effective, even when transported to rather different cultural backgrounds [14,15].

This may be the case for Portugal. Following international trends, cases of childhood behavior problems in Portugal have been consistently rising. In 2023, 17% of cases reported by Portuguese Child Protective Services [16] were related to behavioral problems, such as defiance, aggression, hyperactivity, difficulty following instructions and rules, and challenges in emotional regulation. However, to our knowledge, many Portuguese services do not have any evidence-based responses with which to effectively intervene. Thus, it is critical to provide Portuguese services with an empirical tool that can aid practitioners.

The Being a Parent (Portuguese version: Ser Pai & Ser Mãe) program is an evidence-based parenting program developed in the United Kingdom (UK). It has many advantages, mainly associated with it being a peer-led, brief, group intervention with highly cost-effective results [17]. A significant body of research supports the effectiveness of the Being a Parent program in addressing childhood behavioral issues. It has been extensively evaluated in the UK [18,19] and has also been implemented and assessed in Australia [20], with both evaluations demonstrating promising outcomes in reducing externalizing behaviors and enhancing parental well-being. Furthermore, independent organizations in both the UK and Australia have rated the *Being a Parent* program as an effective and cost-efficient parenting intervention [17,21]. In fact, a meta-analysis from Piquero et al. [22] that compared the Being a Parent intervention with similar interventions currently available suggests that it is one of the most effective group interventions for reducing disruptive behavior and improving positive parenting skills. This effectiveness may stem from its peer-led delivery model, which promotes relatability and trust among participants. Additionally, its content is grounded in the most evidence-based and effective methodologies (e.g., attachment approaches) for group-based parenting interventions targeting childhood behavior problems. A key strength of the program is also its emphasis on parental self-care, equipping parents with tools to enhance their own well-being alongside improving their parenting skills.

Implementing an evidence-based group parenting program, such as the Being a Parent program, in Portugal can have various benefits. Firstly, this program equips parents with practical, evidence-based parenting strategies that promote positive behavior and emotional regulation in children, by promoting strong parent–child relationships, setting clear boundaries, and using positive reinforcement, which are essential tools for managing and reducing behavioral issues [18]. By also focusing on parent self-care, the program helps reduce parental stress and enhances their ability to manage challenging behaviors effectively [18]. Hence, it can address behavior problems in children at an early stage, thus preventing the escalation of externalizing difficulties and reducing the likelihood of more severe problems in the future. Moreover, the positive effects of the Being a Parent program extend beyond families and can impact communities overall, as seen in the national scaling study from Day et al. [19]. Finally, this program is considered a cost-effective tool, making it feasible for implementation in service settings.

Notwithstanding the aforementioned findings, many questions arise from the practicability of effectively implementing the Being a Parent program in Portugal. Indeed, there are substantial contextual disparities between Anglosphere and non-Anglosphere countries, like the UK and Portugal. Consequently, there is a need for a systematic adaptation of the Being a Parent program to take into account linguistic, legal, and cultural factors compatible with the cultural patterns, meanings, and values distinctive to Portugal [23]. For example, one main difference from the UK context concerns the practice of psychology. In Portugal, the practice of psychological acts is regulated and exclusively entrusted to licensed psychologists, limiting the possibility of implementing peer-led approaches. Thus, changes regarding the delivery of the program will have to be executed to adapt the intervention to the Portuguese legal context.

Moreover, it is important to take into consideration that very few evidence-based parenting interventions have been tested in Portugal, and thus, the feasibility of implementing an evidence-based parenting program, such as the Being a Parent program, raises relevant, unanswered questions pertaining to acceptability, feasibility, and effectiveness. Indeed, the acceptability of these types of programs by Portuguese parents, as well as their subjective experiences, have yet to be thoroughly explored. Therefore, it becomes imperative to gain empirical knowledge of these crucial aspects prior to committing to a comprehensive trial. In this sense, conducting a feasibility study is key for the exploration of the impact of cultural differences and parents’ adherence and acceptability, and for the refinement of a subsequent large-scale randomized controlled trial (RCT). Feasibility studies allow practitioners to uncover implementation issues, address uncertainties related to trial methodologies, and assess the initial effects of an intervention [24].

Thus, this paper outlines the methodology and procedures of a feasibility RCT that aims to test the potential impact of the Being a Parent program in the Portuguese context. Our goals are four-fold: (a) to analyze key feasibility parameters (such as adherence and retention); (b) to assess the fidelity and acceptability of the Portuguese version of the Being a Parent program; (c) to explore the effectiveness of the program in childhood behavior problems; and (d) to explore the effectiveness of the program in parenting skills, parental concern, and parental sense of competence.

## 2. Materials and Methods

The Being a Parent parenting program will be tested in Portugal using a two-arm parallel-group feasibility RCT. The following section describes the design, participants, and methodology of this study.

### 2.1. Design

**Figure 1:**
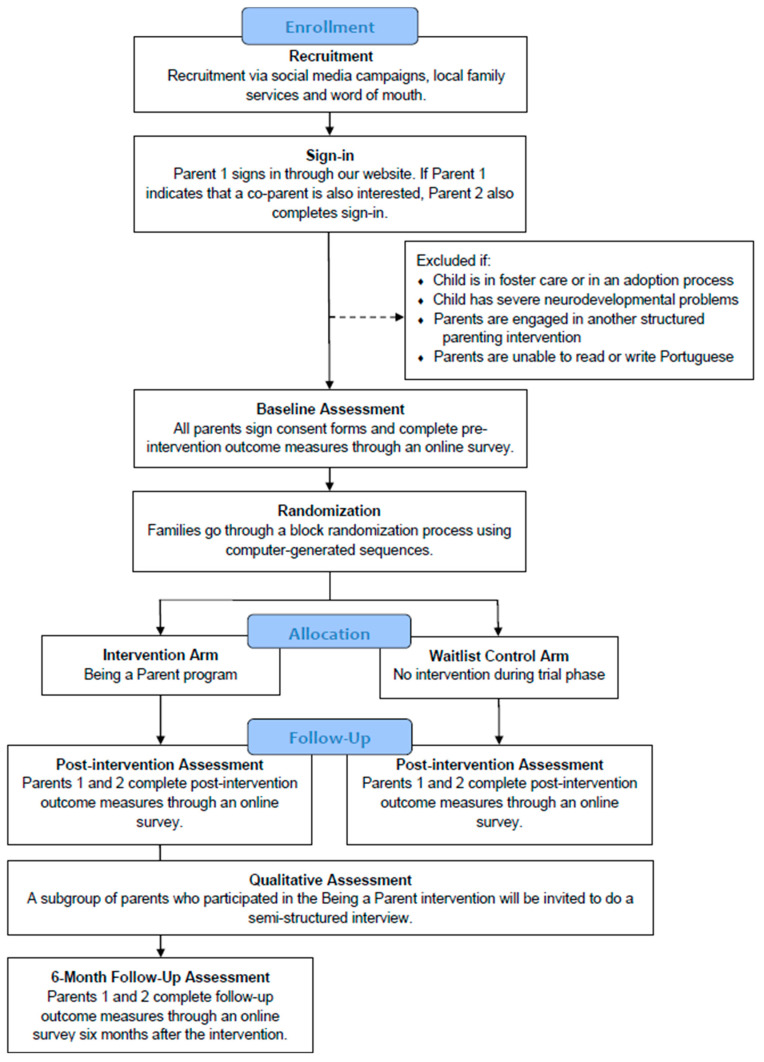
Diagram of participant flow throughout the study.

This is a two-arm parallel-group feasibility RCT, in which families will be randomly assigned to an intervention arm and a waitlist control arm, with a 1:1 allocation ratio. Parents in the intervention group will participate in the Being a Parent program, while the waitlist control group will not receive any intervention during this phase but will be given the opportunity to participate in the program after post-intervention assessments are conducted. Two outcome assessments will be completed by both arms at baseline and post-intervention, and an additional 6-month follow-up will be conducted only with the intervention arm, as assessing the control group at this stage would not provide accurate control data for comparison since the control group will have been participating in the Being a Parent program. Concurrently, a qualitative process assessment will be performed using semi-structured interviews to thoroughly explore the experience of the families who participated in the Being a Parent program, independently of their designated trial group. Figure 1 summarizes the participant flow throughout the study. As illustrated, parents will be recruited through different channels and will be asked to sign in through our website. If included in the study, participants will complete baseline assessments and proceed to randomization to either an intervention group or a control group. Post-intervention assessments will be completed by both groups, and the intervention group will have an additional 6-month assessment. Qualitative assessments will also be conducted.

### 2.2. Participants and Eligibility

The participants will be primary caregivers from the district of Lisbon or Setúbal (Portugal) with a child between the ages of 2 and 11 years old. One or both parents can choose to participate. Families will be eligible for inclusion if a parent identifies difficulties in managing the child’s behavior. Since the Being a Parent program is designed to improve parenting skills for the management of behavioral issues, as long as parents are struggling with child behavior, they are likely to find the intervention helpful. As exclusion criteria, the following principles were defined: (a) families where the index child is in foster care or in an adoption process at the time of the study, as such children are not cohabiting with their parents; (b) parents of children with severe neurodevelopmental problems (such as autism), as children with neurodevelopmental disabilities often require specialized interventions; and (c) parents who are engaged in another structured parenting intervention; and (d) parents who are unable to read or write Portuguese.

### 2.3. Procedures

#### 2.3.1. Recruitment

Participants will be recruited through four main channels, namely, (1) social media, (2) primary schools and children’s centers, (3) social services and child protective services, and (4) health centers, such as hospitals. Prospective parents will be directed to the study’s website for more information and will have access to an online registration survey where they can formalize their interest and sign in. Since both parents from a family unit can participate, when the first parent (Parent 1) registers, they may convey the interest of their co-parent (Parent 2) in participating and provide their details. Subsequently, the research team will contact Parent 2 to officialize their registration.

Following the completion of the registration form, the research team will assess eligibility. Those deemed ineligible will be promptly communicated with and informed of other available community resources. Eligible caregivers will be notified of their eligibility via e-mail, and two weeks before the beginning of the parenting program, they will be asked to complete consent forms and baseline measures, also through an online survey, to formalize their participation in the study. Informed consent will be provided by checking a box in the survey acknowledging that parents have read all the information and agree to participate.

#### 2.3.2. Randomization

The randomization of participants will occur after the collection of baseline data and will be conducted only by one researcher, assuring that the rest of the research team will be blinded to randomization. Participants will be randomly assigned to either the intervention group or the waitlist control group using block randomization to ensure balanced allocation across key variables that could influence the study outcomes. Blocks will be created using Random Allocation Software (version 2.0) and will be based on four stratification variables: child’s age (categorized into predefined age ranges to account for developmental differences), child’s sex (male or female, as behavioral outcomes may vary by gender), socioeconomic status (SES; classified into low, medium, or high, based on parental education), and whether one or both parents of a family unit will participate. Within each block, participants will be randomly assigned to groups using a computer-generated randomization sequence, ensuring equal distribution across intervention and control groups. This approach will minimize confounds by balancing critical baseline characteristics and will enhance the internal validity of the study [25]. Additionally, to ensure transparency and replicability, the randomization sequence and its implementation will be documented in detail and securely stored for auditing purposes.

#### 2.3.3. Data Collection

Outcome quantitative measures will be completed by all parents in two assessment periods, namely, baseline and post-intervention. In addition, participants from the intervention arm will have a third assessment period, namely, a 6-month follow-up. Primary caregivers with multiple children will be asked to designate a target child for whom they will complete the measures. They will be advised to select the child who they have more concerns about in terms of behavioral difficulties. In the cases where both parents from a family unit participate, Parent 1 (i.e., the first caregiver to sign in to the program) will choose the target child. Parent 2 will be notified of the age of the target child by the research team and will be asked to complete the questionnaires while thinking of this child. Researchers will also advise Parent 2 to confirm the name of the child selected as the target with Parent 1.

Measures will be completed through an online survey created on Qualtrics.com and have an estimated completion duration of 20 min. Parents will have a maximum of two weeks to fill out the survey in each assessment period. Qualitative interviews will be conducted post-intervention in person in a research setting. Parents who participated in the Being a Parent program will be asked to take part in the interview and all that accept will be included. The interview will follow a pre-designed guide, consisting of an average of 20 questions and 35 associated probes, to explore parents’ experiences with the intervention and its feasibility (e.g., “*What did you think about the homework assignments*”, “*Did you ever considered quitting the program?*”, “*What were the benefits of the program?*”, “*What are your opinions about the group leaders?*”). This interview will have an average duration of 1.5 h and will be recorded with a recorder owned by the interviewer. Parents will also be advised not to share any personal information that may identify them during the interview.

#### 2.3.4. Incentives

Families will receive monetary incentives (i.e., vouchers) from a national supermarket. Each family will receive a EUR 5 voucher for each assessment completed, regardless of their assigned study arm. Families who complete both the baseline and post-intervention measures will also qualify for entry into a draw for a chance to win a EUR 200 voucher. At the 6-month assessment, families will have the opportunity to enter another draw for a EUR 20 voucher. No monetary incentives will be delivered for completing the qualitative interviews. The primary goal of providing these monetary incentives is to promote the completion of assessment measures.

#### 2.3.5. Ethical Considerations

This project has been approved by the Ethics Committee from Ispa—University Institution [D-054-11-22] and is registered on ClinicalTrials.gov [NCT05626244]. Data collected will be confidential, and parents will be given a unique identification number in order to guarantee that the questionnaires and the interviews do not contain any information that may identify them. Furthermore, in compliance with the General Data Protection Regulation (GDPR), personally identifiable information will be securely stored separately from outcome data. Additionally, surveys and recordings will be stored for a period of five years, after which they will be permanently deleted.

Ethical considerations guided the provision of vouchers, and the prespecified monetary value was deemed sufficient to acknowledge the commitment of families without exerting undue influence on their decisions. Participants will be transparently informed of the incentive structure before informed consent is requested.

### 2.4. The Portuguese Being a Parent Program

The Being a Parent intervention is part of the Empowering Parents, Empowering Communities program developed in the United Kingdom (UK) by the Centre for Parent and Child Support at South London and Maudsley NHS Foundation Trust (SLAM) and the Child and Adolescent Mental Health Services (CAMHS) Research Unit at King’s College London. It consists of eight weekly sessions plus an optional introductory session, each with a duration of two hours, facilitated by two accredited parents of the community and directed at groups of seven to twelve parents [18].

Built on attachment theory, family systems theory and cognitive–behavioral frameworks, it is rooted in empirically validated parenting strategies that specifically address the most common issues present in children aged two to eleven years old with behavioral problems [19]. Each session is characterized by its high degree of interactivity, featuring a dynamic combination of small and large group discussions, role-playing, demonstrations, the sharing of experiences, and reflective exercises [19]. Table 1 describes in detail the content discussed in each session.

Adapting the Being a Parent intervention to the Portuguese community encompassed pertinent cultural and delivery changes. Following a stacked intervention adaptation model, namely, the Ecological Validity Model (EVM) [23,26] and the Cultural Adaptation Process (CAP) [27], the Being a Parent program was translated to Portuguese, and minor changes related to linguistic features were implemented in order to culturally adapt the intervention. No content modifications were made due to the fact that all topics and exercises were considered pertinent to the Portuguese context, despite cultural differences that may exist. Due to legal aspects, the main modification in the Portuguese version was focused on program delivery. Instead of using a peer-led approach, the Portuguese version will be professionally led, meaning that two accredited psychologists with experience in child and familial problems will deliver the intervention.

Moreover, the parenting program will be delivered face to face in a university setting, namely, at Ispa—University Institution (Lisbon). To ensure a comprehensive experience of the program and its processes, a minimum of five members per group will be required to start the intervention. Parents will complete the program and receive a participation certificate if they attend at least six sessions. Babysitting services will be provided in all sessions, allowing parents to bring their child while they attend the program. Two accredited psychologists will deliver the program to all groups.

### 2.5. Control Condition

Families randomized to the control group will be in a waitlist condition. Hence, participants will not receive any active treatment in parallel with the intervention arm but will have the opportunity to subsequently take part in the Being a Parent program.

The choice of the control condition was guided by several considerations. Firstly, to the best of our knowledge, there are no current “standard of care” parenting interventions for childhood behavior problems in Lisbon or Setúbal, and thus, comparing the Being a Parent program with an active control group is not feasible. Moreover, given the limited availability of support in the community, the inclusion of a waitlist condition will enable us to extend intervention to all participating parents.

### 2.6. Measures

#### 2.6.1. Feasibility, Fidelity, and Acceptability

Structured sheets will be used to record significant feasibility parameters associated with trial methodology and program implementation. This will enable the assessment of participants’ rates related to (a) enrolment; (b) eligibility; (c) intervention attendance and retention; and (d) data collection. Dropout will also be recorded for both arms, as well as missed sessions for the experimental group. The research team, with the help of the group leaders, will fill out weekly review forms to assess fidelity criteria (e.g., the delivery of content; alignment with intervention methods and strategies). In addition, a treatment acceptability rating scale (TARS) created by the Empowering Parents, Empowering Communities team will be completed by the participants from the experimental group post-intervention. This scale was specifically created in order to assess the unique aspects of treatment acceptability that are specific to the Being a Parent program. It comprises 12 tailored items in which parents will report on satisfaction with intervention delivery. Questions reflect the content and delivery style of this specific parenting program, ensuring that the assessment is both contextually relevant and comprehensive (e.g., “*In general, are you satisfied with group leaders?*”).

A qualitative semi-structured interview will also be conducted post-intervention with a small sample of the participants from the experimental group. The main goal is to better understand the experiences of parents that participate in the parenting program. Therefore, topic guides will include open prompts regarding (a) trial implementation (e.g., recruitment and data collection); (b) intervention implementation (e.g., localization and dates); (c) intervention acceptability (e.g., content and impact); and (d) the adequacy of group leaders (e.g., competencies).

#### 2.6.2. Psychological Outcomes

The following self-report questionnaires will be completed by parents through an online survey at baseline, post-intervention, and at follow-up. These measures were carefully chosen to provide a well-rounded understanding of the child’s behavioral challenges, parenting styles, and the parents’ sense of competence, all of which are central to the intervention’s goals. All participants will also complete a sociodemographic questionnaire at baseline designed specifically for this trial.

1. Child Behavior Checklist for Preschool and School Years (CBCL) [28,29]. This is a self-report measure that is completed by caregivers of children between the ages of 1.5 and 18 years old [29]. It consists of different statements describing various behavioral and emotional problems, and caregivers must rate the extent to which each statement applies to their child. Only the Externalizing Scale (the Attention Problems, Aggressive Behavior, and Delinquent Behavior in the school years version—33 items) and the subscale Anxiety/Depression (12 items) will be used.
2. Parenting Styles and Dimensions Questionnaire (PSDQ) [30,31]. This is a self-report questionnaire designed to assess different parenting dimensions and three main styles, namely, authoritative, authoritarian, and permissive [31]. Parents must rate their agreement or disagreement with statements about their parenting strategies [31].
3. Parental Concerns Scale [32]. Divided into five concern dimensions, this evaluates main domains of parental concerns, namely, family and school problems; eating, sleep, and physical complaints; preparation; fears; and negative behaviors [32].
4. Parenting Sense of Competence (PSOC) [33,34]. An adapted version for at-risk parents of Parenting Sense of Competence will be used to assess parents’ feelings of competence in their parenting roles [34]. It comprises two subscales, namely, Satisfaction and Efficacy [34].

All measures have been adapted to and validated in Portugal and present adequate psychometric properties.

### 2.7. Sample Size

Sample size calculation was based on the CONSORT 2010 statement extended to feasibility trials [35] and the Lewis et al. [36] methodology. This approach calculates sample size by conducting hypothesis testing based on estimations of predefined feasibility outcomes being within the unacceptable “red zone” (null hypothesis) and contrasting this with an alternative hypothesis of being within the “favorable” green zone [35]. A sample size is considered appropriate if it allows for ≥80% power to reject being in the red zone if the green zone holds true. For detailed information about this methodology, please consult Lewis et al. [36].

Progression parameters and their subsequent point estimates as described in Table 2 were used. Considering a one-tailed, one-sample binomial test based on normal approximation (with continuity correction) with α = 0.05, the following minimum sample sizes for each parameter were obtained:

1. Feasibility of participant retention at post-intervention (Rul = 0.50 and Gll = 0.70)—41 participants
2. Feasibility of consent rate (Rul = 0.40 and Gll = 0.60)—42 participants
3. Feasibility of program completion (Rul = 0.45 and Gll = 0.75)—18 participants (intervention arm)
4. Feasibility of program acceptability (Rul = 0.45 and Gll = 0.75)—19 participants (intervention arm)
5. Feasibility of program fidelity (Rul = 0.40 and Gll = 0.70)—19 participants (intervention arm)

Therefore, a sample size of 42 participants, with 25 participants in the intervention arm, allows for ≥80% power to reject the null hypothesis for each of the feasibility outcomes. Moreover, this sample size is sufficient for the estimation of effect sizes for a future larger trial, as it allows for the collection of preliminary data needed to calculate reliable effect size estimates and assess the variability in psychological measures.

Regarding our qualitative data, according to the recommendations of Boddy [37] and van Risjnsoever [38], we aim to recruit 10 to 12 participants, as this sample size is often sufficient to achieve data saturation when the aim is to explore shared experiences within a relatively homogeneous group. Moreover, we will closely monitor data saturation during analysis and will adjust the sample size if necessary to ensure robust and meaningful findings.

**Table 2:** Feasibility progression parameters.

Parameter	Thresholds		Justification
1. Feasibility of Retention of Families at Post-Intervention *Can a significant number of recruited families be retained until post-intervention?*	GREEN ZONE (Go—Proceed with full RCT)	>70%	Ensuring a substantial post-intervention retention rate is essential to obtain accurate and unbiased post-intervention data Favorable retention rates at the feasibility trial will contribute to estimating the required sample size for a larger RCT Systematic reviews have shown that attrition rates between 9 to 27% can be found in RCTs testing parenting interventions for childhood behavior problems [39] Previous trials of the BaP program reported an average retention rate of 80% [18,19]
	AMBER ZONE (Amend—Proceed with changes)	50–70%	
	RED ZONE (Stop—Do not proceed unless significant changes are made)	<50%	
2. Feasibility of Consent Rate *Is the rate of eligible families who consented and were randomized favorable for a large-scale RCT?*	GREEN ZONE (Go—Proceed with full RCT)	>60%	An adequate consent rate is essential for the feasibility trial to accurately estimate the number of families who need to be reached, in order to achieve the required sample size to obtain significant statistical power in a large-scale RCT. Low consent rates may provide important information regarding issues in recruitment procedures (such as locations of recruitment or information available to interested participants). Previous trials report an average consent rate of 70% [40] Previous trials of parenting interventions in Portugal report consent rates ranging from 63% to 97% [41,42] Previous studies of the BaP program report an average consent rate of 71% [19]
	AMBER ZONE (Amend—Proceed with changes)	40–60%	
	RED ZONE (Stop—Do not proceed unless significant changes are made)	<40%	
3. Feasibility of Program Completion *Can a significant number of participants from the intervention arm complete the BaP program?*	GREEN ZONE (Go—Proceed with full RCT)	>60%	Achieving favorable program completion rates is critical to obtain accurate and unbiased data A systematic review [43] found a mean program completion rate of 80% with a wide variety across studies between 50% and 100% Previous trials of the BaP program have shown an average completion rate of 70% [18,19]
	AMBER ZONE (Amend—Proceed with changes)	30–60%	
	RED ZONE (Stop—Do not proceed unless significant changes are made)	<30%	
4. Feasibility of Program Acceptability *Does an appropriate number of families from the intervention arm rate the BaP program acceptable?*	GREEN ZONE (Go—Proceed with full RCT)	>75%	Program acceptability is an important criterion in considering if the BaP program will be well received in the Portuguese community Program acceptability will be measured using the treatment acceptability rating scale (TARS) A total score of 27 or more equates to ratings of “Quite a lot” or “A great deal” on each item Previous trials of the BaP program found that an average of 98.5% of participants had total scores ≥ 27 [19] A conservative threshold of 75% will be used
	AMBER ZONE (Amend—Proceed with changes)	45–75%	
	RED ZONE (Stop—Do not proceed unless significant changes are made)	<45%	
5. Feasibility of Program Fidelity *Can the BaP intervention be delivered as planned?*	GREEN ZONE (Go—Proceed with full RCT)	>70%	For the BaP program, 75% fidelity is considered appropriate, which would mean that six out of eight sessions were delivered as planned Fidelity estimates serve as indicators to identify deviations from the designed intervention delivery, exposing potential issues like methodological challenges and variances with local contexts [44] Increasing fidelity correlates with improved outcomes [44] Conservative estimates of fidelity rates are used because this is the first time the BaP program will be tested in a Portuguese context, and thus, cultural and linguistic factors can significantly impact fidelity
	AMBER ZONE (Amend—Proceed with changes)	40–70%	
	RED ZONE (Stop—Do not proceed unless significant changes are made)	<40%	

### 2.8. Data Analysis Strategy

#### 2.8.1. Quantitative Data

Quantitative data will be analyzed using IBM SPSS Statistics (version 28). The analysis and presentation of data will be in accordance with CONSORT guidelines, in particular the extension to feasibility and pilot trials [35].

Descriptive statistics will be computed to assess feasibility and acceptability. Fidelity data collected from the weekly review forms will be assessed based on a cumulative score across sessions, as well as a percentage of the maximum cumulative score. Standard descriptive statistics will be computed to report demographics, as well as baseline and outcome scores. An intention-to-treat (ITT) analysis approach will be employed to ensure the validity and generalizability of study findings by minimizing bias and providing a robust assessment of treatment effects [45]. Effectiveness analyses, using data from our psychological measures, will be performed with data from Parent 1, while data collected from Parent 2 will be examined independently to consolidate our findings. An analysis of covariance (ANCOVA) will be used to examine differences between intervention and waitlist control arms, taking pre-intervention data into account as a covariate. Cohen’s d effect sizes will be calculated, as well as 95% confidence intervals. Data from Parent 1 and Parent 2 will be compared using paired-samples sign tests in order to determine if there were any differences in the median values of their observations.

When the assumption of homogeneity of regression is not met, transformations will be performed on pre-intervention data. Moreover, Little’s test [46] will be conducted to examine the underlying nature of our missing data. If missing data are missing completely at random (MCAR), a Last Observation Carried Forward (LOCF) framework will be used.

#### 2.8.2. Qualitative Data

Nvivo (version 14) software will be used to transfer interview transcripts. Data analysis will be an ongoing, iterative process, occurring concurrently with data collection, in order to successively explore emerging themes with participants. A thematic analysis will be conducted to identify common themes that might emerge in regard to parents’ experiences. Moreover, an inductive, semantic approach will be used, and two researchers will independently code transcripts. Any discrepancies will be solved by consensus. Additionally, Cohen’s Kappa will be computed as a measure of inter-coder reliability.

#### 2.8.3. Parameters of Feasibility Success

The parameters to determine feasibility success were based on the Mellor et al. [47] recommendations. Five feasibility parameters were outlined, and three thresholds were created (green, amber, and red zones) to decide if it is feasible to progress to a large-scale RCT. Table 2 describes each parameter and the subsequent threshold values.

## 3. Discussion

This protocol describes an RCT designed to assess the feasibility and potential effectiveness of the Portuguese adaptation of the Being a Parent program. We expect that our findings will be congruent with previous studies of the Being a Parent program [18,19,20], which reported parents’ satisfaction with the intervention and moderate to large effects on childhood behavioral problems, parenting skills, and parenting stress. Moreover, we expect that the Being a Parent intervention in Portugal will be at least as effective when compared to other programs available in Portugal (e.g., Incredible Years) [48].

Notwithstanding, inherent challenges associated with this feasibility study can be identified. For example, randomization can pose challenges in terms of participant retention. Therefore, various strategies (e.g., communication strategies) will be implemented to ensure that families remain involved and committed to the trial. Cultural variables may also surface as significant factors. Although the Being a Parent program is an evidence-based, manualized intervention, further modifications may be necessary to enhance resonance and relevance. It is also important to take into account that this feasibility trial uses a comprehensive approach of data collection, using both quantitative and qualitative methods to assess outcomes. In acknowledgment of the potential time constraints and participant burden associated with this approach, monetary incentives in the form of vouchers will be delivered to families to encourage the completion of measures. In addition, possible logistical and resource constraints may emerge as challenges.

The potential implications of this study are noteworthy. Firstly, this is the first feasibility RCT of the Being a Parent program outside of the United Kingdom. Thus, it represents a pivotal step in assessing the universal strength of the Being a Parent program, especially as it is being implemented in a country with distinct cultural norms and values, as well as diverse political, religious, and service contexts. Secondly, this is the first trial of the Being a Parent program employing a professionally led approach, deviating from the original peer-led methodology. Consequently, the outcomes of this study will offer valuable insights, shedding light on the distinctions between professionally led and peer-led methodologies when implementing the Being a Parent program. Thirdly, this feasibility study holds significant promise as the knowledge that will be acquired will play a crucial role in future strategic and methodological decisions for a full-scale RCT in Portugal. Hence, it will help ensure that a subsequent comprehensive trial is meticulously designed and tailored to effectively address cultural, linguistic, and contextual factors related to Portugal, ultimately enhancing the overall efficacy of the intervention. Moreover, findings from this study will contribute to the refinement of future trials not only in Portugal but also in other countries that share similar cultural backgrounds, thus fostering a more comprehensive and culturally attuned approach to parenting interventions across diverse settings. At last, introducing the Being a Parent program in Portugal will provide services with a cost-effective, evidence-based tool to deal with externalizing behaviors at an early stage and mitigate the risk of the development of more severe problems.

## Figures and Tables

**Table 1:** “Being a Parent” course outline ^1^.

**Session 1: Being a Parent**
Getting to know each other Goals for parent and child ‘Good enough’ vs ‘perfect’ parent Taking care of ourselves
**Session 2: Feelings**
Remembering what it was like to be a child Acknowledging and accepting feelings Expressing feelings
**Session 3: Play**
Child-led play—Special time
**Session 4: Valuing my child**
Avoiding ‘labels’ and describing behavior Using descriptive praise to change behavior
**Session 5: Understanding children’s behavior**
Understanding children’s needs and their behavior in response to needs Discipline Commands, consequences, rewards, and star charts
**Session 6: Discipline strategies**
Understanding what we mean by boundaries Time Out and saying ‘No’ Household rules
**Session 7: Listening**
Communication styles Helping a child when upset ‘Open’ and ‘Closed’ questions Reflective listening
**Session 8: Review and support**
Coping with stress Reviewing the course and knowing where to get support Ending and celebration

^1^ Adapted from Being a Parent: Manual for Course Facilitators (4th ed, p. 12) by C. Penney, C. Wilson, L. Draper, C. Day and C. Kearney, 2018. Copyright 2018 by Caroline Penney, Charlotte Wilson, Lucy Draper, Crispin Day and Catherine Kearney.
